# Study protocol for a randomised controlled trial of diacerein versus placebo to treat knee osteoarthritis with effusion-synovitis (DICKENS)

**DOI:** 10.1186/s13063-022-06715-w

**Published:** 2022-09-11

**Authors:** Guoqi Cai, Graeme Jones, Flavia M. Cicuttini, Anita E. Wluka, Yuanyuan Wang, Catherine Hill, Helen Keen, Benny Antony, Xia Wang, Barbara de Graaff, Michael Thompson, Tania Winzenberg, Kathy Buttigieg, Dawn Aitken

**Affiliations:** 1 Department of Epidemiology and Biostatistics, School of Public Health, Anhui Medical University, Hefei, Anhui China; 2 Menzies Institute for Medical Research, University of Tasmania, Hobart, TAS Australia; 3 Department of Epidemiology and Preventive Medicine, Monash University, Melbourne, VIC Australia; 4 The Queen Elizabeth Hospital, University of Adelaide, Woodville, SA Australia; 5 Discipline of Medicine, University of Adelaide, Adelaide, South Australia Australia; 6 Department of Rheumatology, Fiona Stanley Hospital, Murdoch Drive, Murdoch, WA Australia; 7 Medical School, University of Western Australia, Crawley, WA Australia; 8 Rheumatology Department, Royal North Shore Hospital and Institute of Bone and Joint Research, Kolling Institute, University of Sydney, Sydney, NSW Australia

**Keywords:** Diacerein, Effusion-synovitis, Knee pain, Magnetic resonance imaging (MRI), Osteoarthritis (OA), Randomised controlled trial

## Abstract

**Background:**

There is an unmet need for treatments for knee osteoarthritis (OA). Effusion-synovitis is a common inflammatory phenotype of knee OA and predicts knee pain and structural degradation. Anti-inflammatory therapies, such as diacerein, may be effective for this phenotype. While diacerein is recommended for alleviating pain in OA patients, evidence for its effectiveness is inconsistent, possibly because studies have not targeted patients with an inflammatory phenotype. Therefore, we will conduct a multi-centre, randomised, placebo-controlled double-blind trial to determine the effect of diacerein on changes in knee pain and effusion-synovitis over 24 weeks in patients with knee OA and magnetic resonance imaging (MRI)-defined effusion-synovitis.

**Methods:**

We will recruit 260 patients with clinical knee OA, significant knee pain, and MRI-detected effusion-synovitis in Hobart, Melbourne, Adelaide, and Perth, Australia. They will be randomly allocated to receive either diacerein (50mg twice daily) or identical placebo for 24 weeks. MRI of the study knee will be performed at screening and after 24 weeks of intervention. The primary outcome is improvement in knee pain at 24 weeks as assessed by a 100-mm visual analogue scale (VAS). Secondary outcomes include improvement in volumetric (ml) and semi-quantitative (Whole-Organ Magnetic Resonance Imaging Score, 0–3) measurements of effusion-synovitis using MRI over 24 weeks, and improvement in knee pain (VAS) at 4, 8, 12, 16, and 20 weeks. Intention-to-treat analyses of primary and secondary outcomes will be performed as the primary analyses. Per protocol analyses will be performed as the secondary analyses.

**Discussion:**

This study will provide high-quality evidence to determine whether diacerein improves pain, changes disease trajectory, and slows disease progression in OA patients with effusion-synovitis. If diacerein proves effective, this has the potential to significantly benefit the substantial proportion (up to 60%) of knee OA patients with an inflammatory phenotype.

**Trial registration:**

Australian and New Zealand Clinical Trial Registry ACTRN12618001656224. Registered on 08 October 2018.

## Background

Osteoarthritis (OA) is a highly prevalent, painful, disabling, and costly condition that affects over 250 million people worldwide [1]. Current treatments focus on alleviating pain because patients rank pain control as their highest treatment priority [2]. Despite this, pain control remains poor, with >75% of patients reporting the need for additional symptomatic treatment [3]. Furthermore, despite the large disease burden of OA, there are currently no approved disease-modifying drugs available to prevent or stop the joint damage. OA is a complex, heterogeneous disease with multiple phenotypes; these will require different approaches for each patient to optimise treatment [4–7]. The overall lack of treatment efficacy for OA may be due to treating everyone as if they have the same pathological process.

Localised inflammation is recognised as an important factor in OA pathogenesis and is a catalyst for joint deterioration [4]. Inflammation in the knee joint is visualised as increased signal intensity on T2-weighted magnetic resonance imaging (MRI) which results from inflammation of the joint lining (synovitis) and joint fluid (effusion), termed “effusion-synovitis”. Effusion-synovitis is present in approximately 60% of OA patients [8] and correlates well with histological features of inflammation [9]. In population-based studies, effusion-synovitis predicts increases in knee pain in a dose-response manner [10] and is associated with cartilage damage, bone abnormalities, cartilage loss [11], and joint replacement [12]. Therefore, stopping the cascade of inflammation has the potential to reduce pain, slow or stop deleterious changes in knee structure, and delay joint replacement in OA patients.

Pro-inflammatory cytokines such as interleukin-1α and β (IL-1α and IL-1β), interleukin-6 (IL-6), and tumour necrosis factor-α (TNF-α) play major roles in the development of OA symptoms as well as disease progression [9]. IL-1 presents in multiple joint tissues of OA [13], including the cartilage, synovial membrane, and fluid [9]. Preclinical and clinical studies provide significant evidence for the role of IL-1 in OA pathogenesis, including cartilage degradation, subchondral bone remodelling, and synovial proliferation and inflammation. Members of the IL-1 family stimulate neutrophils, which play a key role in producing acute synovitis in OA patients [14]. IL-1 also stimulates chondrocytes and synoviocytes which adversely promote cartilage destruction in OA, as well as inhibit the synthesis of proteoglycan and collagen type II, the main components of articular cartilage [14]. Injection of IL-1 into animal knees results in cartilage loss [15] and blocking its activity leads to a reduction in OA progression [14]. IL-1 knockout mice are resistant to surgically induced cartilage damage and development of inflammation and pain, compared to wild-type mice [14]. In a gene expression study in OA patients using peripheral blood leukocytes, those with an overexpression of IL-1β had higher pain scores, decreased function and a 3-fold increased risk of x-ray progression over 2 years [16]. In summary, IL-1 expression in OA plays a key role in synovitis and is associated with more severe disease, including pain and rapid disease progression.

Diacerein is a semisynthetic anthraquinone derivative that blocks IL-1β, and in vitro, also stimulates the production of cartilage growth factors such as transforming growth factor β [17]. In animal models of OA, diacerein significantly reduced cartilage degradation compared with untreated animals [18, 19]. In humans, the few acceptable-quality trials show inconsistent results concerning the effect of diacerein in OA. The pooled results from the latest systematic review of 10 trials showed that diacerein had a small beneficial effect on pain and joint space narrowing (JSN) [20], but the quality of evidence was low. Apart from their relatively low quality, another limitation of existing trials is that they include patients with knee OA regardless of whether they had an inflammatory phenotype. As diacerein targets inflammation, it may be that its effects will be greater and more definitive if its use is targeted to patients with signs of inflammatory OA.

Recommendations for the use of diacerein for OA treatment are inconsistent. Although the European League Against Rheumatism (EULAR) [21]) has recommended diacerein as a treatment option for OA, updated international guidelines from the Osteoarthritis Research Society International (OARSI) [22] and the American College of Rheumatology (ACR) [23] recommend against its use, in part because of concerns regarding adverse events, most notably diarrhoea. However, both the European Medicines Agency (EMA’s) Pharmacovigilance and Risk Assessment Committee (PRAC) [24] and the European Society for Clinical and Economic Aspects of Osteoporosis and Osteoarthritis (ESCEO) [25] have evaluated the efficacy and safety of diacerein and concluded that the benefit of diacerein outweighs its known risks for OA treatment. A chief investigator on the present study also performed a review and concluded that diacerein is safe, has a modest effect on pain, and is a worthwhile treatment option for OA [13]. Thus, the use of diacerein for OA treatment should not be ruled out due to safety issues.

Therefore, we aim to conduct a multi-centre, randomised, double-blind, placebo-controlled trial, to determine the effect of diacerein on knee pain and effusion-synovitis over 24 weeks in patients with clinical knee OA, significant knee pain, and MRI-detected effusion synovitis.

### Objective

We are conducting a multi-centre, randomised, double-blind, placebo-controlled trial aiming to compare the effect of diacerein vs. placebo on knee pain and effusion-synovitis over 24 weeks in 260 knee OA patients with effusion-synovitis. We hypothesise that diacerein will improve knee pain over 24 weeks (primary hypothesis) and decrease effusion-synovitis over 24 weeks (secondary hypothesis) compared with placebo. We also hypothesise that diacerein will be more effective in patients with moderate to severe effusion-synovitis (secondary hypothesis).

## Methods

### Study design

This protocol is reported as per the Standard Protocol Items for Clinical Trials (SPIRIT) Statement [26]. The diacerein for knee osteoarthritis with effusion-synovitis (DICKENS) study is a multi-centre, randomised, double-blind, placebo-controlled superiority trial over 24 weeks. This trial has been developed according to the OARSI Recommendations for conducting clinical trials for knee OA [27] and a consensus for conducting and reporting OA phenotype research [28]. The trial was registered (Australian and New Zealand Clinical Trial Registry, ACTRN12618001656224) prior to recruitment, and trial reporting will be guided by the Consolidated Standards of Reporting Trials (CONSORT) Statement [29] and the CONSORT and SPIRIT Extension for RCTs Revised in Extenuating Circumstances (CONSERVE) implementation tool [30]. This trial will also be run in accordance with the National Health and Medical Research Council (NHMRC) Statement, *the Coronavirus disease 2019 (COVID-19): Guidance on clinical trials for institutions, HRECs, researchers and sponsors* *[*31*].* We aim to recruit 260 patients with clinical knee OA, significant knee pain and effusion-synovitis present on MRI. To recruit the patients, we will use an established strategy [32], including collaboration with general practitioners, specialist rheumatologists, orthopaedic surgeons, and advertising through local media, social media (e.g. Facebook, Twitter and University websites), contacting past knee OA trial participants, patient support groups and hospital flyers. Patients will be recruited from four sites (i.e. Hobart, Melbourne, Adelaide, and Perth) within Australia, and each site aims to recruit 65 participants. Ethics approval has been obtained from the Tasmania Health and Medical Human Research Ethics Committee (H0017151), Monash University Human Research Ethics Committee (17684), Alfred Hospital Ethics Committee (427/18), and South Metropolitan Health Service Human Research Ethics Committee (RGS0000000957). Written informed consent or eConsent (through an online survey issued using Research Electronic Data Capture (REDCap)) will be obtained from all patients by a medical doctor at each site. The consent or eConsent form includes two optional permissions, (1) consent for their medical records to be made available to study researchers from the Australian Orthopaedic Association National Joint Replacement Registry (AOANJRR) for the purpose of checking on any knee joint replacement surgery following study completion and (2) consent to have blood samples stored for future testing and to share blood samples and associated data with other researchers, including researchers outside Australia.

### Inclusion criteria

1. Males and females aged 40 to 64 years (as currently recommended for diacerein treatment by the EMA’s PRAC [24]).
2. Significant knee pain (defined as ≥ 40mm on a 100mm visual analogue scale (VAS)) on most days in the past month.
3. Meet ACR clinical criteria for knee OA [33] confirmed by a medical doctor.
4. Any knee effusion-synovitis present on MRI: Presence will be defined as any effusion (Grade 1–3), using the modified Whole-Organ Magnetic Resonance Imaging Score (WORMS) scoring system [10, 11]. During screening effusion-synovitis will be scored from 0 to 3: 0 = no effusion-synovitis in the joint (patients will not be eligible for this trial); 1 = < 33% of maximum potential distention; 2 = 33–66% of maximum potential distention; 3 = > 66% of maximum potential distention.
5. Participants who are screened via Telehealth must have radiographic knee OA defined as joint space narrowing or an osteophyte present (score ≥1 on the OARSI atlas [34, 35]).
6. Are willing to participate in the study for 6 months.

### Exclusion criteria

1. Inability to provide informed consent.
2. Contraindication to MRI scanning (for example, implanted pacemaker, metal sutures, presence of shrapnel or iron filings in the eye, claustrophobia, knee too large for scanner).
3. Severe knee OA (defined as Grade 3 JSN on X-ray using the OARSI atlas [34, 35]) as the potential for any treatment to have benefit is considered very small in this group. Old films will be accepted up to 2 years before the screening date.
4. Other forms of arthritis (e.g., rheumatoid arthritis, gout or other inflammatory arthritis).
5. Significant injury in the study knee within the last 6 months.
6. Arthroscopy or open surgery in the study knee in the last 12 months.
7. Received intra-articular therapy (e.g. corticosteroids, hyaluronic acid) in the study knee in the last 6 months.
8. Planned arthroscopy or joint replacement surgery during the study period.
9. Contraindication to diacerein use including:
  - Patients with a known tendency towards diarrhoea.
  - Patients with inflammatory bowel disease (e.g. Crohn’s disease, ulcerative colitis).
  - Patients who have stomach problems with unknown cause.
  - Patients who are taking a diuretic medication or heart failure medication (digitalis glycoside).
  - Patients who have a current and/or history of liver disease. A screening blood test will be performed to assess liver function. Patients with abnormal liver function will be excluded (defined as alanine transaminase (ALT) 2x upper normal limit, normal range for ALT is 7–55 units per litre (U/L) therefore an ALT of ≥110 U/L will be defined as abnormal).
  - Patients with abnormal kidney function (creatinine clearance < 30 ml/min).
  - Patients with a known hypersensitivity to this sort of medication, i.e. anthraquinone derivatives (includes some laxatives (dantron, emodin, aloe emodin and some senna glycolsides), antimalarials, and antineoplastics used in the treatment of cancer (mitoxantrone, pixantrone, and the anthracyclines).
  - Patients who are having ongoing antibiotic and/or chemotherapy treatment.
  - Patients who are lactose intolerant, as the study medication contains lactose.
  - Women who are pregnant or breastfeeding.
10. Use of any investigational drug(s) and/or devices within 30 days or 5 half-lives (whichever is longer) prior to randomisation.

### Randomisation and blinding

Allocation of patients in a 1:1 ratio to either the active or placebo group will be by computer-generated random numbers using a central randomisation website hosted by the University of Tasmania. We will use block randomisation (permuted block design), using a block size of 4 (2 in each arm), stratified by study site and size of effusion-synovitis (Grade 1 or 2/3). This will be conducted by a staff member with no other involvement in the study.

The randomised controlled trial will be a double-blind one, with study patients, assessors, and MRI readers all blinded to treatment allocation. Allocation concealment and double blinding will be ensured by (1) the use of identical capsules for each group; (2) objective measures of knee structural changes being made by trained observers blinded to group allocation; and (3) subjective measures being taken by research assistants blinded to group allocation.

Emergency unblinding will be allowed in limited situations that impact on the safety of study participants. Code-break for the full randomisation schedule will be maintained by the administering institute. Participants who are unblinded will be withdrawn from treatment but will continue to be followed as per the planned follow-up schedule.

### Intervention

The treatment dose for this trial is diacerein 50 mg twice daily, as this is the recommended therapeutic dose for this drug. Eligible patients will start the trial taking one capsule daily with food, containing 50 mg of diacerein or identical placebo, for the first 2 weeks. This will then be increased to two capsules daily with food, equating to 100 mg of diacerein or identical placebo, to be taken for the remainder of the 24-week trial. The gradual increase in dose, as recommended by PRAC [24], aims to reduce the side effect of loose stool and diarrhoea caused by diacerein.

At 2 weeks, study staff will contact participants by telephone to assess any potential side effects and instruct them to increase dosage if appropriate. Depending on the side-effect profile participants report during the trial, it is possible for participants to remain on 50 mg/day after week 2 or for participants to reduce their dose from 100 mg/day back to 50 mg/day anytime during the trial. This decision will be made in consultation with the medical doctor at each site. The reason participants can remain on half the dose is that the effect of diacerein on pain improvement does not appear to be dose-responsive. For example, the literature suggests that 50 mg/day has a similar efficacy compared to 100 mg per day (− 15.6 vs − 18.3) [36].

### Study procedure and time points

Research assistants will first conduct telephone screening. Recruitment documentation will be posted to all individuals who satisfy telephone screening. Potential participants will have at least one week after receiving their recruitment documentation to read and consider a face-to-face visit or Telehealth screening. Patients will undergo, in sequential order, (i) clinical assessment with a medical doctor, as well as blood tests to ensure no contraindication to diacerein and a urine test in premenopausal women to rule out pregnancy, (ii) x-ray to confirm radiological disease and exclude severe knee OA, (iii) MRI of the study knee to determine presence of effusion-synovitis. If both knees are symptomatic and meet the ≥40mm/100 VAS inclusion criteria, then the knee with the worse pain and mild JSN will be studied.

For participants who are screened via Telehealth, the physical knee examination will be replaced by a verbal discussion with a medical doctor, talking through the ACR criteria. The study doctor will use the results of the verbal Telehealth knee exam, the participants’ medical history and medications, along with the participants’ x-ray and MRI scans to confirm the OA diagnosis and ensure it is safe for the participant to be enrolled in the study being guided by the current inclusion/exclusion criteria. During the Telehealth screening appointment, if something concerning comes up which requires face-to-face screening, the study doctor can either request the participant attend in person or exclude this participant if necessary.

Table 1 outlines the schedule of assessments. There will be 9 assessments (screening, weeks 0, 2, 4, 8, 12, 16, 20, and 24), and face-to-face clinic visits or Telehealth appointments will occur at screening, weeks 0, 12 and 24. The same research nurses, who are blinded to treatment allocation, will measure all clinical variables, administer questionnaires, monitor compliance, and record adverse events at these visits. Telephone contact and/or mail-outs (by email) will occur at the other time-points (weeks 2, 4, 8, 16, and 20). MRI scans will occur at screening and week 24; knee x-ray will be performed at screening.

**Table 1:** Schedule of assessments

	Screening		Double-blind period							
	Screening Clinic visit or Telehealth	Post-screening	Baseline (week 0) Clinic visit or Telehealth	Week 2	Week 4	Week 8	Week 12 Clinic visit or Telehealth	Week 16	Week 20	Week 24 Clinic visit or Telehealth
Informed consent^a^	x									
Knee x-ray^b^		x								
Bloods (safety and storage)^c^		x					x			x
Urine^d^	x									
Knee MRI		x^e^								x
Randomisation			x							
**Clinic visit or Telehealth**	x		x				x			x
ACR clinically diagnosed OA	x									
Leg strength			x^f^				x^f^			x^f^
Height and weight			x^g^				x^g^			x^g^
Capsules dispensed			x^h^				x^h^			
Dosage increase				x						
Capsule count (adherence)							x^i^			x^j^
**Questionnaire measures**	x		x		x	x	x	x	x	x
Demographics (sex, DOB)	x									
Medicare number			x							
Knee VAS	x		x		x	x	x	x	x	x
WOMAC pain/function/stiffness			x		x	x	x	x	x	x
OMERACT-OARSI Responder criteria			x		x	x	x	x	x	x
Knee surgery and injections	x		x		x	x	x	x	x	x
Medication use	x		x				x			x
Safety (adverse events)				x	x	x	x	x	x	x
AQoL-8D and EQ-5D-5L			x				x			x
Health economics outcomes: *Medication cost diary* *Health service utilisation* *Employment/days off work* *Concession/Health Care Card* *Private health insurance* *Transport and specialised equipment costs*			x							x
painDETECT			x				x			x
Depression			x				x			x
Fibromyalgia-ness			x				x			x
Treatment guessing							x			x
Consent to be contacted for future studies										x
Early withdrawal information				As required						

^a^To be performed/reviewed by the study doctor

^b^To exclude severe knee OA (defined by bone on bone present on x-ray using the OARSI atlas [34]) or to ensure radiographic knee OA is present for Telehealth Screening Appointments only. Old films will be accepted up to 2 years ago

^c^Blood tests will be performed at screening, weeks 12 and 24 to exclude participants with abnormal liver and kidney function. Blood samples will also be stored for future biomarker testing

^d^Performed only in premenopausal women where there is a possibility of pregnancy

^e^Knee MRI performed to determine the presence of effusion-synovitis. MRI is only performed once the participant meets all other inclusion/exclusion criteria (i.e. x-ray and blood test results meet inclusion/exclusion criteria)

^f^Will not be assessed/recorded during Telehealth appointments

^g^Will be self-reported if possible, during Telehealth appointments

^h^Participants must complete the safety pathology testing at screening to be enrolled in the study and dispensed study medication. Safety pathology testing is also required at week 12 before the study medication is dispensed

^i^During Telehealth appointments, participants will be provided with a reply-paid envelope to return their unused study medication for capsule counting

The COVID-19 pandemic may limit face-to-face visits due to local restrictions or distance considerations. In this case, patients will have the option of Telehealth appointments, and survey data will be completed as per usual via REDCap. The physical measurements that take place at these visits will be omitted or substituted as appropriate (see outcome measures for detail).

### Quality assurance

To ensure high-quality execution of the trial in accordance with the protocol, all trial staff will be trained by the chief investigators and provided with a standard protocol book (with details of standard operating procedures used, trial contacts, visits, measurements, and monitoring) and case report forms.

### Outcome measures

#### Primary outcome measure

The primary outcome measure is improvement in knee pain at 24 weeks, as assessed using a 100mm VAS. Patients will be asked “On this line, thinking about your *right/left* knee, where would you rate your pain, using the last 7 days as a timeframe”.

#### Secondary outcome measures

##### Improvement in effusion-synovitis at 24 weeks

A non-contrast MRI scan of the study knee will be performed at screening and week 24 using 1.5-T or 3-T whole-body MRI units with a commercial transmit-receive knee coil. For each participant, the study knee will be imaged in the sagittal plane using the same scanner at each study site. The following sequences will be used: (1) T2-weighted fat-saturated 3D fast spin echo sequence and (2) T1-weighted fat-saturated 3D gradient-recalled acquisition sequence (Table 2).

**Table 2:** Magnetic resonance imaging sequences and parameters at the four study sites

	Machine and coils	T2-weighted sagittal 3D	T1-weighted sagittal 3D
Hobart	1.5 T whole-body MR unit (GE Optima 450W, Milwaukee, USA), using a dedicated Transmit/Receive 8-channel knee coil where patient size permits, if body habitus is too large, we use a 16-channel large GEM Flex coil	T2 weighted fat-saturated 3D fast spin echo sequence; flip angle 90°, repetition time 2300 ms; echo time 95 ms; field of view 18 cm; 256 × 256 matrix; 2 excitations; slice thickness 1 mm	T1-weighted fat-saturated 3D gradient recalled acquisition; flip angle 30°; repetition time 38 ms; echo time 3 ms; field of view 16 cm; 256 × 256 matrix; 1 excitation; slice thickness 1.5 mm
Melbourne	3 T whole-body MR unit (Siemens, Magnetom Avanto Fit, Erlangen, Germany), using a dedicated Transmit/Receive 15 channel knee coil	T2 weighted fat-saturated 3D fast spin echo sequence; flip angle 120°; repetition time 1200 ms; echo time 50 ms; field of view 16 cm; 256 × 320 matrix; 1 excitation; slice thickness 0.6 mm	T1-weighted fat-saturated 3D gradient recalled acquisition; flip angle 30°; repetition time 11.7 ms; echo time 5.61 ms; field of view 16 cm; 320 × 320 matrix; 1 excitation; slice thickness 1.5 mm
Perth	1.5 T whole-body MR unit (Siemens, Magnetom Avanto Fit, Erlangen, Germany), using a dedicated Transmit/Receive 15 channel knee coil	T2 weighted fat-saturated 3D fast spin echo sequence; flip angle 90°; repetition time 2302 ms; echo time 81 ms; field of view 18 cm; 256 × 256 matrix; 2 excitations; slice thickness 1 mm	T1-weighted fat-saturated 3D gradient recalled acquisition; flip angle 30°; repetition time 17.7 ms; echo time 3.5 ms; field of view 18 cm; 256 × 160 matrix; 1 excitation; slice thickness 1.4 mm
Adelaide	3 T whole-body MR unit (Siemens, Magnetom Skyra 3Tesla), using a dedicated 15-channel knee coil	T2 weighted fat-saturated 3D SPACE; flip angle 120°; repetition time 2200 ms; echo time 121 ms; field of view 16 cm; 320 × 259 matrix; 1 excitation; slice thickness 0.6 mm	T1-weighted Water Excitation 3D gradient recalled acquisition VIBE; flip angle 16°; repetition time 10.4 ms; echo time 5.7 ms; field of view 160 cm; 320 × 320 matrix; 1 excitation; slice thickness 1 mm

Effusion-synovitis volume (mL) will be distinguished in the following subregions according to the anatomy of the knee joint synovial cavity [37]: (1) the suprapatellar pouch, extending superiorly from the upper surface of the patellar, between the posterior suprapatellar fat pad (quadriceps femoris tendon) and the anterior surface of the femur; (2) other cavity, which includes the area between the central femoral and tibial condyles, around the ligaments and menisci, and the area behind the posterior portion of each femoral condyle, inside of the joint capsule. The volumes of individual joint subregions will be isolated from the total volume by selecting each region of interest (ROI) according to the intra-articular fluid-equivalent signal on a section-by-section basis. The final 3-dimensional volume rendering will be generated using commercial in-house imaging software. The readers will be blinded to treatment allocation and participant information. Intra- and inter-observer repeatability for this measurement method is excellent, with an intraclass correlation coefficient (ICC) of 0.95–0.98 and 0.93–0.99, respectively [8]. Change in total effusion-synovitis volume and volume at each sub-region will be calculated as follow-up volume minus baseline volume.

Effusion-synovitis in each subregion will also be scored individually according to modified Whole-Organ Magnetic Resonance Imaging Score (WORMS), grading collectively from 0 to 3 based on the estimated maximal distention of the synovial cavity: 0 = no effusion-synovitis in the joint; 1 = <33% of maximum potential distention; 2 = 33–66% of maximum potential distention; 3 = > 66% of maximum potential distention [38]. Total effusion-synovitis score of the whole joint will be defined as the maximum score of each subregion, ranging from 0 to 3. Changes of total effusion-synovitis score and score at each sub-region will be calculated by subtracting the baseline score from the follow-up score, and a change in score of ≥1 will be defined as an increase. The inter-rater reliability of this method in our hands was 0.63–0.75 and intra-reader reliability was 0.60–0.75 (weighted kappa) in different subregions as described previously [11].

##### Improvement in knee pain at 4, 8, 12, 16, and 20 weeks

Change in knee pain, measured by a 100mm VAS and the proportion of participants improving by the minimum clinically important difference (15mm) [39] at weeks 4, 8, 12, 16, and 20 are secondary outcomes.

##### Change in pain, function, and stiffness over 24 weeks

WOMAC (Western Ontario and McMasters Universities Osteoarthritis Index [40]) is a widely used instrument in OA research and recommended by OARSI [27] which assesses knee pain, function and stiffness. Improvements in WOMAC knee pain, function, and stiffness will be assessed over 24 weeks.

##### OMERACT-OARSI responder criteria

A simplified set of responder criteria focused on pain, function and patient global assessment developed by OMERACT (Outcome measures in Rheumatology)-OARSI will be assessed at all time-points [41].

##### Co-pathology present on MRI

Bone marrow lesions (BMLs) defined as an ill-defined hyperintensity in the subchondral bone, on MRI will be assessed on the sagittal T2 weighted sequences at the medial tibial, medial femoral, lateral tibial, lateral femoral and patella sites by means of image processing on commercial in-house imaging software. The maximum size of each lesion will be measured in mm^2^ using software cursors applied to the greatest area of the lesion, as previously described [32]. Previously we have demonstrated an ICC of 0.84 to 0.91 [32], using this method. Total BML size (mm^2^) will be calculated as the sum of every lesion within the medial tibial, medial femoral, lateral tibial, lateral femoral and patella sites at screening and 24 weeks.

Cartilage defects will be assessed at the medial tibial, medial femoral, lateral tibial, lateral femoral and patella sites using modified Outerbridge classification system, as we have previously described [42]: grade 0 = normal cartilage; grade 1 = focal blistering and intra-cartilaginous low-signal intensity area with an intact surface and base; grade 2 = irregularities on the surface or base and loss of thickness <50%; grade 3 = deep ulceration with loss of thickness >50%; and grade 4 = full-thickness chondral wear with exposure of subchondral bone. In our previous study, we demonstrated the ICCs ranged from 0.80 to 0.95 [42] for the different knee sites, using this method.

Meniscal extrusion will be assessed as we have previously described [43] as the proportion of the menisci affected by a partial or full extrusion at the anterior, middle, and posterior horns (medially/laterally). In our previous study we demonstrated the intra- and inter-reader ICCs ranged from 0.85 to 0.92 for meniscal extrusion [44].

##### Lower limb muscle strength

Lower limb muscle strength is a key correlate of pain and tends to increase when pain is reduced [45]. We will assess leg strength by dynamometry at the lower limb (involving both legs simultaneously) at weeks 0, 12 and 24. The muscles measured in this technique are mainly the quadriceps and hip flexors. The previously published repeatability estimate (Cronbach’s α) for this method is 0.91 [46]. This measure will be omitted at Telehealth appointments.

##### Knee surgery and joint injections

Whether the participant undergoes any knee surgery (including arthroscopies or joint replacement surgery) or injectable treatment during the trial, will be assessed by questionnaire at each time point. Study participants will also be asked to provide consent to have their data linked to the Australian Orthopaedic Association National Joint Replacement Registry (AOANJRR). *Any data linkage that occurs as part of this study will be subject to conditions around data security and privacy for our participants. The data that we receive from other databases will only be used for the purposes outlined in our study protocol.*

##### Concomitant medication use

Medication usage (including prescription, over-the-counter, and natural/herbal remedies) will be documented at each clinic visit (screening, baseline, 12 and 24 weeks). Participants will be asked to keep analgesic medications as stable as possible but if there are changes to the medications used or dose changes during the trial the reason will be documented.

##### Health economics outcomes

Health state utility, resource utilisation and costs will be measured throughout the study. Health state utility values (HSUV) will be assessed using The Assessment of Quality of Life (AQoL-8D) [47] and the 5-level EuroQoL-5 dimensional version (EQ-5D-5L) [48] at baseline, weeks 12 and 24. OA-related health service use will be assessed at baseline and week 24. This will include visits to primary care physicians, medical specialists, and physiotherapists; medical imaging, procedures, tests and investigations for knee pain; hospital admissions and attendances for knee pain, and use of community services related to knee pain. Patient incurred costs for transport and specialised equipment purchased/hired due to knee pain will also be collected. For services subsidised by Medicare, costs will be taken from the Medicare Benefits Schedule; patient out-of-pocket costs will be self-reported.

Indirect costs will include absenteeism and presenteeism due to knee pain. Participants will be asked about their concession, health care card & private health insurance status as costs vary based on these.

##### Bloods

Blood samples will be taken at screening, weeks 12 and 24 for safety and storage. The safety blood tests performed at each time point include a full blood count, urea and electrolytes test (UEC) and a liver function test. Storage of blood samples will occur at each time point for future biomarker testing. Potential biomarkers to be tested include inflammatory markers, cartilage and synovial degradation markers which have been implicated in the pathogenesis of knee OA. The blood will be stored at − 80°C.

##### Treatment guessing

At 12 and 24 weeks, participants will be asked what treatment they think they received with the following options: Active treatment (diacerein), Inactive treatment (placebo), or Not sure.

##### Adherence

At 12 and 24 weeks, participants will be asked to return all bottles (used or unused), such that adherence can be calculated based on pill counts [49]. All bottles (both returned and un-dispensed) are to be stored on site until the next Monitoring Visit. After reconciliation, returned bottles will be destroyed on-site.

#### Additional measures

##### Demographics and Medicare number

At screening we will collect information on sex and date of birth. At baseline we will record participants’ Medicare number for the purpose of future data linkage. Medicare is the publicly funded universal health care insurance scheme in Australia that fully or partly covers treatments and services provided by health practitioners.

##### Anthropometrics

Height (stadiometer) and weight (electronic scales) will be measured at weeks 0, 12 and 24. BMI will be calculated as weight/height^2^. During Telehealth appointments, height and weight will be self-reported if possible.

##### X-ray

The degree of X-ray damage may be an effect modifier. A standing anteroposterior semi-flexed x-ray of the study knee will be performed at screening for classification purposes. X-rays will be scored for joint space narrowing and osteophytes on a four-point scale (0–3) using the OARSI atlas [34, 35]. This method has very high reproducibility with an ICC of 0.98 for joint space narrowing and 0.99 for osteophytes [50].

##### PainDETECT

The painDETECT questionnaire assesses to what degree a participant’s pain is neuropathic like [51]. This may be an effect modifier and will be measured at weeks 0, 12 and 24.

##### Depression

Depression may be an effect modifier. It will be assessed using the Patient Health Questionnaire (PHQ-9) [52] at weeks 0, 12 and 24.

##### Fibromyalgia-ness

We will use the Symptom Impact Questionnaire (SIQR) to assess patients’ symptoms of fibromyalgia which is a validated questionnaire to assess fibromyalgia-ness in non-fibromyalgia patients [53, 54]. This will be assessed at weeks 0, 12 and 24.

### Safety

The risks associated with diacerein use have been well-documented [24]. Adverse events (AEs) will be monitored throughout the study. Standard safety and efficacy monitoring will be performed through regular face-to-face visits and/or Telehealth appointments and/or phone calls between visits. The patients are requested to report any AE to the research staff spontaneously. Details of the AE and its relationship with study intervention will be recorded and reported to the Ethics Committees. We will code all the AEs according to the Medical Dictionary for Regulatory Activities (MedDRA).

### Sample size calculations

#### Pain (primary outcome)

Based on in-house data from the 4Jointz trial [45], 234 participants will give us 90% power, with 5% probability of type 1 error (alpha=0.05) to detect a 10mm difference between diacerein and placebo on the VAS pain scale (standard deviation (SD) of pain change 25.5 in the placebo group and 21.5 in drug group). To allow for a 10% loss to follow-up, we need 260 participants (130 in each arm). We expect to see a larger effect than published in the diacerein meta-analysis (8.7mm) [20] due to targeting the therapy for those with effusion-synovitis. We will have >99% power to detect a difference of 15 mm, the minimum clinically important difference (MCID), on a 100mm VAS for OA trials [39].

##### Pre-specified stratification analysis based on size of effusion-synovitis (secondary analysis)

One hundred twenty-two participants at the end of the trial with Grade 2 or 3 effusion-synovitis will give us 80% power, and 5% probability of type 1 error (alpha=0.05) to detect a 12mm difference between diacerein and placebo on the VAS pain scale (SD of pain change 25.5 in the placebo group and 21.5 in drug group) in this participant sub-group. Based on the distribution of effusion-synovitis in our vitamin D trial for knee OA [55], we expect to recruit approximately 145 participants with either Grade 2 or 3 synovitis.

#### Effusion-synovitis (secondary outcome)

With 234 subjects, we will have over 99% power to detect a difference of 4.5ml, based on a SD of change of 7.44 in the placebo group and 6.74 in the drug group. These SDs have been estimated from our vitamin D trial for knee OA [8] using data from participants with Grade 1 or higher effusion-synovitis and a VAS score ≥ 40mm. We expect to see at least a reduction of 4.5 ml effusion-synovitis if diacerein effectively targets inflammation. While an MCID for effusion-synovitis is uncertain, a 4.5-ml reduction is approximately 2.5 times the change that could be expected with measurement error (±1.81mL [8]).

### Statistical analysis

The primary analyses will be intention-to-treat analyses of primary and secondary outcomes. Per protocol analyses will be performed as the secondary analyses, for study participants consuming ≥80% of study medication between baseline and week 24 (allowing for 1 capsule (50 mg) per day).

Changes in pain scores and total effusion volume will be analysed using a linear mixed-effects model with treatment, month and their interaction (treatment × month) as covariates. The correlation within trial centres and the repeated measures will be addressed using the trial centre and participant identification as random intercepts. Month will be treated as a random effect to allow different treatment effects among participants over time. Change in outcome measures within each group and difference of the changes between groups from baseline to follow-up will be calculated using linear combinations of the estimated coefficients adjusted for the baseline values of the corresponding outcome measure (e.g. change in pain scores will be adjusted for baseline pain scores). We will also run a model that additionally adjusts the primary outcome for sex, analgesic medication, and depression. Missing data caused by loss to follow-up and nonresponses will be addressed by adding baseline complete variables that can explain the missingness to the regression models.

Secondary analysis for missing data will be performed using multiple imputation by chained equations, with 20 imputations performed by the treatment group using baseline complete variables and nonmissing values of the outcomes at baseline and each follow-up, assuming missing at random.

The steering committee recommended that a doubling of serious adverse events (SAE) in one group compared to the other will trigger a safety review by the Data and Safety Monitoring Board (DSMB). If a safety review is triggered, the DSMB will review the unblinded data, and make recommendations about stopping the trial. A death (or other significant SAE) in the drug group may lead to stopping the study. When the DSMB reviews the unblinded data, they will make a judgement about whether the death/SAE is clearly attributable to the drug, and this will inform the decision about stopping the trial. No interim analyses will be conducted for efficacy. As this is a trial for OA (a non-life-threating condition), no level of efficacy would outweigh SAE concerns. An extraordinary meeting of the DSMB may be called by the Steering Committee at any time if there is concern about the number or severity of AEs.

Based on our hypothesis that diacerein will be more effective in participants with moderate to severe effusion-synovitis, we will perform a stratified analysis based on the size of effusion-synovitis at baseline (mL), and ordinal effusion-synovitis score (Grade 1 or 2/3) at baseline. Other pre-specified stratification analyses will be performed to examine which subgroups may respond better to treatment based on these variables: radiographic knee OA severity, degree of neuropathic pain, depression, fibromyalgia-ness, and co-pathology present on MRI. Statistical significance will be set as a two-sided *P* value <0.05.

### Data integrity and management

All data will be collected using REDCap electronic data capture tools hosted at the University of Tasmania. Paper copies of participant questionnaires will be stored in locked filing cabinets, with restricted access. Electronic data will be kept on password-protected servers, separating the identifying and non-identifying information. The codes linking data to identifying participant information will be kept separately from the study data, under password protection and with restricted access. Daily backups of all electronic data will occur to minimise any risk of lost data. Only members of the research team who need to contact study participants, enter data or perform data quality control will have access to identifiable information.

After study completion, paper copies of data will be archived in secure storage. Identifiers will not be removed, in case of follow-up of study participants being necessary, but the electronic data will continue to be kept in a secure electronic database, separating identifying and non-identifying information. This will remain password protected and with access given only to the study investigators unless otherwise authorised by the study team.

### Withdrawal

If participants withdraw from the study before 24 weeks of follow-up, the reason and date will be recorded in the Withdrawal Form. Diacerein remains effective for at least two months after treatment is stopped [20]; therefore, to minimise missing data in this study, participants that withdraw from treatment will be asked if they are willing to complete any or all of the remaining assessment items, and any consent given will also be noted in the Withdrawal Form. Participants who choose to withdraw from the trial will be asked permission for the continued use of their blood samples (and data). These participants will be invited to do a second MRI scan as soon as possible, and to complete clinic visits and surveys as they come due until 24 weeks.

### Roles and responsibilities and monitoring

The University of Tasmania (as the trial sponsor) and the principal investigators are responsible for all aspects of the trial, including design, conduct and oversight. The principal investigators will monitor the conduct and progress of the project at each site. The trial coordinator will visit each study site (face-to-face or remotely) to make sure that all trial procedures are compliant with the trial protocol. The principal investigators and the research team will have regular teleconferences to ensure efficient study execution and ongoing monitoring of the study progress, with summary documents circulated after each meeting. An independent data and safety monitoring board will be convened, consisting of 3-5 members, with at least one independent clinical rheumatologist, a clinical pharmacologist, and a biostatistician, all with clinical trial experience. They will monitor adverse events and will meet biannually and provide a written report to the study investigators.

### Dissemination plans

The results of this study will be presented at conferences and published in scientific journals. Any notes or publications arising from our research will de-identified. Only aggregate statistical results will be presented.

The outcomes of the project will be disseminated to participants in an individual letter in non-technical language. The scientific paper will be available for dissemination to patients should they wish to receive it, after the manuscript has been accepted for publication. Dissemination of the overall study findings to the participants will occur in a de-identified manner and be based on the entire study population. No post-trial care will be conducted as there is no anticipated harm and compensation for trial participation.

### Protocol amendments

Protocol amendments include inclusion criteria, dose of intervention, and statistical analysis plan (Table 3). All these changes have been approved by the medical doctors on this study, Data and Safety Monitoring Board, steering committee, and ethics committees.

**Table 3:** Protocol amendments

	Original protocol (version 1.3, 17 September 2018)	Updated protocol (version 1.6, 1 October 2020)	Reasons
Inclusion criteria	1. Males and females aged 40 to 64 years old. 2. Significant knee pain on most days (defined as a visual analogue scale (VAS) greater than or equal to 40mm). 3. Meet American College of Rheumatology (ACR) clinical criteria for knee osteoarthritis confirmed by a rheumatologist. 4. Any knee effusion-synovitis present on MRI. 5. Are willing to participate in the study for 6 months.	One inclusion criterion added: Participants who are screened via Telehealth must have radiographic knee osteoarthritis defined as joint space narrowing or an osteophyte present (score ≥1 on the OARSI atlas).	The protocol has been updated to accommodate Telehealth visits. This is to minimise the impact of COVID-19 on recruitment and retention. Our preference will continue to be face-to-face clinic visits; however, if this is not possible, a Telehealth option will be available. All participants screened via Telehealth will have to have osteoarthritis confirmed on an x-ray, to minimise the risk of enrolling a patient without the condition of interest.
Dose of intervention	Participants will start the trial taking one capsule daily with food, containing 50 mg of diacerein, for the first 2 weeks. This will then be increased to two capsules daily with food, equating to 100 mg of diacerein, to be taken for the remainder of the 24-week trial.	Participants will be allowed to reduce their dose from 100 mg/day to 50 mg/day anytime during the trial after consulting with the medical doctor at each site.	Some participants have experienced side effects that prohibit them from increasing their dosage at week 2. Moreover, the effect of diacerein on pain improvement does not appear to be dose-responsive. For example, the literature suggests that 50 mg/day has a similar efficacy compared to 100mg per day (− 15.6 vs − 18.3) [37].
Statistical analysis	1. If there are baseline imbalances between treatment groups, we will consider adjusting for them based on whether we regard the imbalance as clinically significant. 2. Per protocol analyses will be performed as the secondary analyses, for study participants consuming ≥80% of capsules.	1. We will adjust for the baseline values of the corresponding outcome measure (e.g. change in pain scores will be adjusted for baseline pain scores). We will also run a model that additionally adjusts the primary outcome for sex, analgesic medication, and depression. 2. Per protocol analyses will be performed as the secondary analyses, for study participants consuming ≥80% of study medication between baseline and week 24 (allowing for 1 capsule (50 mg) per day).	For item 1, the trial statistician, along with the data safety monitoring board and steering committee recommended some minor changes to the statistical analysis plan, to adhere to best practice in RCT analysis. For item 2, this is an update to reflect the change in dose of intervention (see above).

## Discussion

We propose a multi-centre, randomised, double-blind, placebo-controlled trial to determine the effect of diacerein on knee pain and effusion-synovitis over 24 weeks in patients with clinical knee OA, significant knee pain, and effusion-synovitis. If diacerein proves effective in patients with moderate to severe effusion-synovitis, it will offer an important therapeutic approach for this inflammatory phenotype of knee OA.

Diacerein is an anti-inflammatory drug that blocks IL-1β, a key marker of inflammatory OA. IL-1 presents in OA joint tissues and plays a key role in synovitis, and the expression of IL-1 is associated with more severe pain and rapid progression of OA. The findings of previous studies suggest that diacerein had a beneficial effect on both knee pain and JSN assessed by x-ray [20]. Considering the mechanism of action of diacerein, it is likely that diacerein has a stronger effect on pain and structural progression in patients with joint inflammation, as indicated by MRI-detected effusion-synovitis. Moreover, by quantitative and semi-quantitative evaluation of effusion-synovitis, this study will determine whether diacerein performs better in patients with more effusion-synovitis, which may reflect a greater component of pain being explained by joint inflammation.

The COVID-19 pandemic is ongoing and will affect study sites recruiting for this trial. To comply with the official rules on restrictions, distancing, and quarantines, and the National Health and Medical Research Council (NHMRC) Statement, *COVID-19: Guidance on clinical trials for institutions, HRECs, researchers and sponsors* [31]*, this study will apply Telehealth*, a technology of digital information and communication. This is an amendment to the original trial protocol, with approval from the ethics committees. The modifications have been described according to the CONSERVE implementation tool [30]. Rather than entirely based on face-to-face visits for screening and follow-up measurements, Telehealth will be an alternative tool in this trial. It is true that Telehealth is not as straightforward as face-to-face visits, but we have set additional inclusion criteria for patients screened via Telehealth, and face-to-face screening is required in the case that Telehealth screening cannot determine the inclusion of patients. Moreover, in such a randomised design clinical trial, patients in both study arms have an equal chance to be screened and followed up via Telehealth. Therefore, we do not foresee that Telehealth will influence the reliability of this study.

In summary, knee OA is a major but poorly understood public health problem with limited treatment options for pain, and no approved disease-modifying drug. Previous studies suggest that diacerein will improve pain and slow structural progression in patients with OA, with the subgroup most likely to benefit being those with an inflammatory phenotype [20]. If diacerein can reduce knee pain and effusion-synovitis in knee OA, study findings will strengthen the use of diacerein in treatment guidelines for OA and be easily translated into clinical practice as diacerein is relatively cheap, well tolerated and currently accessible in Australia and other countries.

## Trial status

Protocol version 1.6 (1 October 2020), updated from version 1.3 (17 September 2018) and approved by ethics committees. This protocol was not submitted earlier because we adapted it due to COVID-19, introducing the ability for Telehealth enrolment and participation. This resulted in some protocol amendments, so we choose not to publish the protocol until these were resolved and we were confident the trial was not going to be abandoned. Upon submission, this study is in the process of patient recruitment. Date recruitment began: 18 March 2019; anticipated completion date of recruitment: 31 July 2022.

## Data Availability

The data that will be generated from this study will not be deposited in a public repository due to privacy and consent restrictions. De-identified data can be made available from the corresponding author on reasonable request, subject to a data sharing agreement.

## Abbreviations

ACR: American College of Rheumatology
AE: Adverse event
ALT: Alanine transaminase
AOANJRR: Australian Orthopaedic Association National Joint Replacement Registry
AQoL-8D: The 8-Dimensional Assessment of Quality of Life
BMI: Body mass index
BMLs: Bone marrow lesions
CONSERVE: CONSORT and SPIRIT Extension for RCTs Revised in Extenuating Circumstances
CONSORT: Consolidated Standards of Reporting Trials
COVID-19: The coronavirus disease 2019
DICKENS: Randomised controlled trial of diacerein to treat knee osteoarthritis with effusion-synovitis
EQ-5D-5L: 5-level EuroQoL-5 dimensional version
EMA: European Medicines Agency
EULAR: European League Against Rheumatism
ICC: Intraclass correlation coefficient
IL: Interleukin
JSN: Joint space narrowing
LDL: Low-density lipoproteins
MCID: Minimum clinically important difference
MedDRA: Medical Dictionary for Regulatory Activities
MRI: Magnetic resonance imaging
NHMRC: National Health and Medical Research Council
OA: Osteoarthritis
OARSI: Osteoarthritis Research Society International
OMERACT: Outcome Measures in Rheumatology
PHQ-9: Patient Health Questionnaire
PRAC: Pharmacovigilance and Risk Assessment Committee
REDCap: Research Electronic Data Capture
ROI: Regions of interest
SAE: Serious adverse events
SIQR: Symptom Impact Questionnaire
SD: Standard deviation
SPIRIT: Standard Protocol Items for Clinical Trials
TNF-α: Tumour necrosis factor-α
UEC: Urea and electrolytes test
UTN: Universal Trial Number
VAS: Visual analogue scale
WOMAC: Western Ontario and McMasters Universities Osteoarthritis Index
WORMS: Whole-Organ Magnetic Resonance Imaging Score
